# The need to study human milk as a biological system

**DOI:** 10.1093/ajcn/nqab075

**Published:** 2021-04-08

**Authors:** Parul Christian, Emily R Smith, Sun Eun Lee, Ashley J Vargas, Andrew A Bremer, Daniel J Raiten

**Affiliations:** Johns Hopkins Bloomberg School of Public Health, Program in Human Nutrition, Department of International Health, Baltimore, MD, USA; Milken Institute School of Public Health, The George Washington University, Departments of Global Health and Exercise and Nutrition Sciences, Washington, DC, USA; The Bill and Melinda Gates Foundation, Seattle, WA, USA; Eunice Kennedy Shriver National Institute of Child Health and Human Development, National Institutes of Health, Bethesda, MD, USA; Eunice Kennedy Shriver National Institute of Child Health and Human Development, National Institutes of Health, Bethesda, MD, USA; Eunice Kennedy Shriver National Institute of Child Health and Human Development, National Institutes of Health, Bethesda, MD, USA

**Keywords:** human milk, nutrients, bioactives, lactation, infant, breastfeeding

## Abstract

Critical advancement is needed in the study of human milk as a biological system that intersects and interacts with myriad internal (maternal biology) and external (diet, environment, infections) factors and its plethora of influences on the developing infant. Human-milk composition and its resulting biological function is more than the sum of its parts. Our failure to fully understand this biology in a large part contributes to why the duration of exclusive breastfeeding remains an unsettled science (if not policy). Our current understanding of human-milk composition and its individual components and their functions fails to fully recognize the importance of the chronobiology and systems biology of human milk in the context of milk synthesis, optimal timing and duration of feeding, and period of lactation. The overly simplistic, but common, approach to analyzing single, mostly nutritive components of human milk is insufficient to understand the contribution of either individual components or the matrix within which they exist to both maternal and child health. There is a need for a shift in the conceptual approach to studying human milk to improve strategies and interventions to support better lactation, breastfeeding, and the full range of infant feeding practices, particularly for women and infants living in undernourished and infectious environments. Recent technological advances have led to a rising movement towards advancing the science of human-milk biology. Herein, we describe the rationale and critical need for unveiling the multifunctionality of the various nutritional, nonnutritional, immune, and biological signaling pathways of the components in human milk that drive system development and maturation, growth, and development in the very early postnatal period of life. We provide a vision and conceptual framework for a research strategy and agenda to change the field of human-milk biology with implications for global policy, innovation, and interventions.

## Introduction

Exclusive breastfeeding is recommended for the first 6 mo of life (1) due to its link to lower infant morbidity and mortality (2). It is promoted through large-scale programmatic action worldwide. While breastfeeding is the targeted behavior of public health interest, human milk is the biologically active delivery system known to benefit the newborn to meet his/her nutritional needs, to provide immuno-protection during the critical period of his/her immature immune system, and to also promote his/her development and gut maturity. Importantly, breastfeeding may benefit the mother's health as well (3). As we learn more about human milk, it is clear that it is more than just food for an infant; it is a biological system with interacting components that affects and is affected by interactions with both the mother and the child. Throughout the article, human milk refers to that biological fluid produced by humans for humans. It can be mother's own milk (MOM) fed directly via breastfeeding or expressed and fed via a bottle. Human milk also includes donor/banked milk. Donor/banked milk contains many of the properties of MOM but is not the same; given the circumstances, it can be used to augment MOM or in cases where MOM is not available.

For the infant, early initiation of breastfeeding and exposure to colostrum (within the first hour of life) affords survival benefit (4). Breastfeeding beyond 6 mo supports both the immune and nutritional status of infants, especially in settings where complementary foods are inadequate and dietary diversity is poor. While the quality of human milk, including milk volume and some nutrients, is widely believed to be maintained even under conditions of mild to moderate maternal undernutrition (5), our knowledge for many micronutrients and bioactives is limited and maternal nutrition and health are likely essential contributors to successful breastfeeding practices and outcomes for both mother and child (6).

Despite its importance in advancing human health, there are major limitations in our understanding of human milk. Nutritional requirements for lactating women are higher than for nonlactating women, and most are even higher than for pregnant women (7). These nutrient reference values for lactation were established for healthy populations, despite limited data on human-milk composition. Accurate information on human-milk composition, with optimal ranges by geography and ethnicity, remains limited. We also have a limited understanding of the relation between human-milk volume and nutrient composition, which may differ by nutrient. In contexts where maternal diets are inadequate and endemic undernutrition and infection are common, it is highly plausible that the nutritionally demanding period of breastfeeding that follows may result in suboptimal lactation unless maternal nutrition is supported. Currently, there are no global policies for nutritional support of the postpartum mother, likely due to an evidence gap in this space. Increasingly, data on growth faltering in early life, including suboptimal thriving of low-birth-weight or preterm infants, also point to the need to design approaches to optimize the health and nutrition of the vulnerable mother–infant dyad (8).

Successfully addressing these scientific and related clinical practice and public health data gaps demands an ecological approach to the study of human milk as a biological system. We need a new conceptual approach to valuing and studying human milk in order to *1*) map the various nutritional and bioactive components of milk by different stages of lactation and their function and mechanism of action for promoting infant growth, development, and survival; *2*) ascertain reference ranges for nutrient levels during early, mid, and late lactation in order to evaluate milk quality and infant feeding practices worldwide; and thereby *3*) enable the design of safe and efficacious interventions for lactation support for women, particularly those living in undernourished and environments with high infectious disease burden. The translation of this emerging knowledge will benefit us in several ways. It will allow us to better define nutrient reference values for both lactating women and their infants, further refine our understanding of the timing and composition of complementary foods, develop integrated strategies to support the development of safe and efficacious human-milk substitutes for infants who do not have access to MOM (either via breastfeeding or expression), and develop a deeper understanding of the optimal role of donor/banked human milk. Furthermore, as we learn more about human milk as a complex system, we better understand the potential implications for lactation success for the mother (e.g., the potential impact of expressing on either human-milk composition or volume) as well as the impact on the infant (e.g., differences between human milk expressed at different times of the day or differences between the use of donor milk and expressed MOM).

Here we outline an existing and future research agenda to enhance our understanding of human milk as a unique biological system in the following sections:

- Understanding human-milk composition as a biological system, including functions of human milk components in promoting newborn and infant health.
- Identifying factors that influence human-milk composition and biology.
- Current and emerging new interventions affecting human-milk composition and infant outcomes.
- Advances in new tools and methods for human-milk analysis and assessment of milk volume.

**FIGURE 1:**
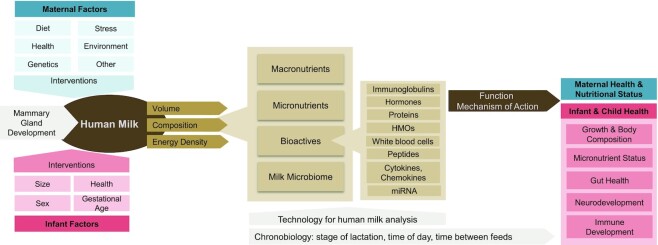
Human-milk research: conceptual framework. HMO, human-milk oligosaccharide; miRNA, microRNA.

**Figure 1** illustrates the key concepts that require investigation, including components in human milk, factors influencing these, and biological mechanisms through which human milk affects human health described below.

## Understanding Human-Milk Composition as a Biological System

Human milk has proven to be the critical link between nutrition and the health and development of infants. We understand it as a source of a number of key nutrient and bioactive elements needed by the infant, but what do we know about its ontogeny, the influence of maternal and infant inputs, and the nature of the relationships between the myriad components that make up this “living” biological secretion?

The unique composition of the biofluid called milk, secreted by the mammalian species catering to their offspring's need, is a result of millions of years of an evolutionary process and in *Homo sapiens* linked to physiologic delays of the immune and even the gastrointestinal system (9).

### Mammary gland development

The biology of the mammary gland, a complex exocrine organ, and the hormonal and other signaling pathways controlling its development, has been delineated (10). Three distinct stages that begin during the embryonic period and occur across the life span, including the pubertal and reproductive adult period, have unique attributes and are controlled by different pathways. Nascent structures established at birth and continued morphogenesis in the postnatal period marks the first stage. Puberty involves further differentiation and branching that results in a functional mammary gland, requiring growth hormone, estrogen, and insulin-like growth factor 1 (IGF-1). The generation of alveoli for milk secretion occurs during pregnancy via the action of both progesterone and prolactin. The final stage is that of involution at weaning, a period during which the mammary gland is remodeled to its prepregnancy state. Because many nutritional factors influence endocrine production, it is plausible that macro- or micronutrient status may influence structural development and function of the mammary gland. For example, vitamin A and retinoid signaling may have a role in its morphogenesis (11). Environmental factors may also influence mammary gland physiology; several studies show endocrine-disrupting compounds and chemicals as well as exposure to heavy metals in gestation to adversely impact these (12). Moreover, a woman's response to environmental and psychosocial stressors may influence both mammary gland development and function (13).

### Milk components

There is a growing body of evidence about the specific components of human milk and the mechanisms through which they influence infant health, growth, and development (**Table 1**) (6, 14–29). The nutritional properties of human milk comprising macro- (e.g., fat, protein, carbohydrate) and micronutrients (vitamins, minerals, amino acids, fatty acids) has been the major focus of study over the past few decades, although very few studies have measured the micronutrient content of human milk or whether micronutrients have unique metabolic fate and function in infants.

**TABLE 1:** Nutritive and nonnutritive components of human milk

	Examples and established or hypothesized function in newborn health
Nutrients	
Water	Human milk meets all water requirements for exclusively breastfed infants.
Carbohydrates	Lactose and HMOs are the most common, but there are many complex carbohydrates in human milk (13).
Protein	There are >400 proteins that have a wide range of functions (13). There are 3 broad categories: casein, whey, mucin proteins [in the milk-fat globule membrane (MFGM)].
Lipids	Packaged as milk-fat lipid globules, lipids account for about half of the energy found in human milk (14, 15).
Micronutrients	Vitamins and minerals are essential for human growth, development, and functioning, and human milk is the only or primary source of micronutrients for exclusively or predominantly breastfed infants (5). They can be grouped into those whose concentrations in human milk are affected by maternal intake or status (type I) or those that are not (type II) (16).
Bioactives	
Immunoglobulins	Secretory IgA (sIgA) (most predominant) and others provide immunologic protection to the infant through antimicrobial, anti-inflammatory, and immunomodulatory mechanisms (13).
Hormones	Leptin and ghrelin are hypothesized to influence infant weight gain and linear growth.
Proteins	Lactoferrin and osteopontin are 2 better-studied bioactive proteins, although there are many. Lactoferrin has been linked to cell growth, iron absorption, and bacteriostatic activities (17–19). Osteopontin (OPN) is thought to play an important role in bone and cartilage development, cell regeneration, and immune system regulation (20).
Human-milk oligosaccharides (HMOs)	The third most common component of human milk (13), which function as prebiotics, antiadhesives, and antibacterials that shape the infant gut microbiome (21)
White blood cells	Historically, maternal/human-milk-derived leukocytes are the most studied cell type and were originally thought to account for the majority of cells in human milk (22). Contemporary work has described their role in conferring active immunity (23) and in making antimicrobial peptides (24).
Antimicrobial peptides (AMPs)	AMPs are short chains of amino acids that offer defense against microbial threats. Broadly, they are a diverse set of molecules in terms of form and exact function.
Cytokines, chemokines	Cytokines in human milk are described for their anti-inflammatory and immune-enhancement properties as well as growth-promoting action (25, 26).
microRNAs	Immunomodulatory component hypothesized to be a key mechanism through which the infant immune system develops (28).
Commensal bacteria	The source and function of commensal bacteria in human milk is an active area of research. *Streptococcus* and* Staphylococcus* are among the most common groups (29). Strains such as *Bifidobacterium* are leading candidates for probiotic supplementation.

Recent breakthroughs in our understanding of the nonnutritional, bioactive, and interactive factors of human milk have revealed a complex “nonnutrient” biologic system that includes an entire immune system, including immune-modulating compounds (27), a system promoting gut maturity [e.g., human-milk oligosaccharides (HMOs)] including growth factors and hormones, and a signaling system involving cell-free RNA in exosomes and microvesicles that may influence infant immunity and microbiota ( 30). The new human-milk paradigm will be informed by our emerging understanding of the potential interactions of nutrients within specific human-milk systems, the role and nature of the human-milk “nutriome,” and its linkages with the nonnutrient bioactives in human milk. Bioactive proteins in human milk provide essential amino acids to infants, appear to be mother-specific, and have multiple functional benefits for infants. Growing evidence suggests specific short- and long-term health consequences linked to lactoferrin, human-milk lysozyme, and osteopontin (18–21, 31–35). HMOs are of the most abundant components of human milk, surpassed only by lipids and lactose. HMOs are hypothesized to be the specific component of human milk that explains why preterm infants fed human milk, as compared with formula, are much less likely to develop necrotizing enterocolitis; research has focused on the specific HMOs (e.g., disialyllacto-N-tetraose) that may be responsible for this health benefit (36, 37). Human-milk hormone concentrations of leptin, ghrelin, and others are clearly linked to energy balance regulation in adults. They have recently been implicated in the physical growth of breastfeeding infants, although data are currently limited and inconsistent (38–40).

Importantly, expanding our understanding of the functions of human-milk components and their roles in newborn and infant health will also affect the translation of that knowledge into evidence-informed guidance regarding the following: modes of infant feeding (breast vs. bottle), optimal timing of introduction and composition of complementary feeding, strategies for feeding low-birth-weight and preterm infants, the safety and efficacy of donor/banked human milk in different populations, and the composition and use of human-milk substitutes.

### The chronobiology of human milk

Milk constituents are highly variable between women but also differ by stage of lactation; the milk that is secreted in the first 4–5 d, called colostrum, is high in carotenoids, in electrolytes such as calcium and sodium, and immunoglobulins and other proteins, but is low in lactose and fat. From about day 5–10 the milk that is secreted is called transitional milk. Mature milk starts being secreted after about 10 d, when lactation is fully established. The fat content of milk changes over time, with colostrum having a lower percentage of fat, relative to transitional milk, whereas mature milk has, on average, 3.6% fat in humans, which may vary somewhat based on time of day, diet, and duration of lactation, but even the length of time elapsed between feeds (14). For example, vitamin concentrations in milk have been shown to vary with time within feed and circadian rhythm (41), and supplementation increases both fat (such as vitamins A and D) and water-soluble (vitamins B-1, B-2, B-12, B-6, and C and pantothenic acid) vitamins. Vitamin concentrations vary dramatically by country in different surveys, and yet, data on optimal levels are lacking and adequate intakes for infants and lactation are based on sparse data with small studies with sample sizes as small as 3 for vitamin A to about 23 for niacin (Institute of Medicine and FAO/WHO) (6). A recent review of vitamin A in human milk described the retinol:fat and retinol concentrations were highest in colostrum, declined dramatically in early lactation, and became stable by 2–4 wk of lactation (42). Each nutrient may have similar increases or decreases by stage of lactation (43). The association between milk volume and concentration of nutrients is not well understood, although energy density is influenced by the fat component of milk, which, in turn, may be influenced by maternal diet, especially of essential free fatty acids.

## Identifying Factors That Influence Human-Milk Composition and Biology

In order to demystify the human-milk system and to better define a translational research agenda, it is critical that we understand and collect information on various maternal and infant factors and the underlying environment including the sociocultural milieu and behavioral norms that influence it. While there is a significant knowledge gap, below is a brief summary of what is currently known regarding determinants.

### Maternal factors

It is assumed that once lactation is established infant demand is the main driver of milk synthesis and production (44). It is generally thought that milk quantity and quality are preserved (5), and infant growth and development sustained, across a range of maternal nutritional states due to physiologic plasticity (45, 46). However, there are many conditions that result in high variability in human-milk composition (47), especially in undernourished settings and sometimes at the cost of depleting maternal stores. Erick (47) describes this phenomenon by saying, “Breast milk is conditionally perfect.” Less appreciated and poorly understood are maternal genetics and modifiable factors such as diet and the environment, for which there is ample experimental evidence in animals but limited data in humans (12). Genetic variants in large milk proteins including α-lactalbumin and α-casein in humans have been found, which, in turn, may be associated with milk volume and composition, as shown in animal studies (12). Genetic variation may also exist for nutrient-specific genes and influence milk composition; 1 or more polymorphisms in vitamin D receptors are associated with milk calcium composition, and mutations in the gene encoding a zinc transporter may influence concentrations of zinc in human milk.

Maternal diet and nutritional status and their influence on lactation were recently summarized (42). Water-soluble vitamins, thiamin, riboflavin, vitamins B-6 and B-12, and choline are known to be affected by maternal diet and/or status, although for most nutrients the data are inadequate. Mineral concentrations are low in human milk and may be stable mainly due to active transport (48). For example, iron in the form of lactoferrin is low in human milk. Zinc concentration in colostrum is high and 17 times higher than in the maternal circulation, both showing preferential transfer and its importance to newborn development (49); in contrast, maternal selenium status influences human-milk selenium concentration (50). Upregulation of calcium absorption in lactation, conservative excretion, and mobilization of bone calcium to maintain calcium concentrations in milk have been reported, mechanisms triggered by endocrine factors and resulting in loss of maternal bone mineral density (51). Iodine concentration in human milk varies widely between populations and gradually declines over the course of lactation (52).

Other lifestyle and environment factors that influence lactation also include exposure to toxins and chemicals (12), alcohol, smoking, vegetarianism, and contraceptive use (53). Postpartum infection and serum interferon-γ have been shown to be significant predictors of milk secretory IgA (sIgA) (54). Overweight and obesity in lactation have been linked to delayed secretory activation and early cessation, in part related to prolactin resistance and reduced signal transducer and activator of transcription 5 (STAT5) activation and decreased insulin sensitivity that may influence milk volume (12). In addition, systemic inflammation related to obesity may also be associated with an inflammatory state in the mammary gland marked by increased proinflammatory cytokines in milk and perturbations in zinc metabolism and lipid synthesis (12).

### Infant factors

In addition to the myriad maternal inputs that influence human-milk composition, infant factors are also important. In particular, the bidirectional “cross-talk” among the microbiota and their metabolites in the infant oral cavity and human milk serves as a conduit for direct signaling from the infant to the mother (55, 56). Moreover, the physical act of suckling, with its attendant dynamic modifications in intraductal pressure within the mammary gland, has implications for human-milk composition. Although not well understood, there appear to be sex-specific interactions in milk composition as related to the influence of maternal factors (57), although data are limited. For example, the protein concentration of milk has been observed to differ by birth mode among male but not female newborns (57).

## Evidence for Interventions and Levers for Influencing Human Milk and Infant Outcomes

### Maternal interventions

Few interventions exist for influencing human-milk composition, despite strong evidence from the dairy and breeding animal literature that shows that dietary inputs can optimize for milk volume, density, and nutrient composition. Maternal supplementation with micronutrients has been examined, although evidence for some nutrients is lacking as is the impact of multiple micronutrient supplementation, which is well tested in the context of prenatal use (58, 59). Little is known about effective dose, frequency of dosing, and timing (stage of lactation) for supplementation. A comprehensive review of the effect of individual micronutrient supplementation on human-milk concentration finds evidence for water-soluble B-vitamins, including thiamin, riboflavin, vitamins B-6 and B-12, and choline, although data are deemed insufficient and, in some instances, the effect is seen only among deficient women (42). Fat-soluble vitamin (vitamins A, D, E, and K) supplementation affects human-milk concentration; postpartum high-dose supplementation was recommended by the WHO for vitamin A-deficient contexts based on evidence of improvements in human-milk concentrations as well as infant status. Mineral supplementation (iron, zinc, copper) has not been shown to influence human-milk composition, although data for these nutrients are sparse. On the other hand, iodine and selenium supplementation may increase human-milk concentration, although again, more data are needed (42). Studies have shown that women who are supplemented with high-dose or daily iodine have increased concentrations of iodine in milk, and to some extent in a dose-responsive way (52). Milk calcium, despite being derived in part by bone remodeling, is not influenced by calcium supplementation, although it may afford some protection to bone mineral accretion in the postpartum period (60).

The link between fatty acid composition of maternal diet and fatty acids in milk is strong. Interventional studies have tested full-fat dairy products, α-linolenic acid (18:3*n*−3), prenatal DHA supplementation, and coconut oil for their effects on human-milk content; these interventions increased the fatty acids in human milk that were provided by the diet or supplement (61). Red palm oil increased provitamin carotenoids in human milk. Several studies using fish-oil supplements have shown an increase in the concentrations of long-chain PUFAs (LC-PUFAs) including DHA and EPA. Fish-oil supplementation in pregnancy has also been associated with higher concentrations of LC-PUFAs in human milk in early lactation (61). Congruent to these findings is the positive dose-dependent correlation between maternal fish consumption and milk DHA. Systematic reviews of intervention studies have consistently identified the gap in our knowledge and understanding of interventions in promoting optimal composition of nutrients in human milk, in part related to the lack of basic understanding of reference ranges for these in healthy mother–infant dyads. We identify a series of research undertakings aimed at addressing this gap at a global level.

### Infant interventions

Based on preclinical work and epidemiological evidence that breastfed infants have a lower risk of morbidity and mortality, several randomized trials have assessed the potential benefits of supplementing formula-fed infants with bovine-based immunomodulatory proteins such as lactoferrin, lysozyme, and osteopontin. Interventions of bovine lactoferrin supplementation among infants in Australia being fed breast-milk substitutes improved infant height and weight gains (62). Similarly, a randomized trial among young children (12–36 mo) in Lima Peru found that 0.5 g lactoferrin/d (as compared with 0.5 g maltodextrin control) improved growth (63). There has been 1 trial among formula-fed infants showing that supplementing with bovine osteopontin reduced the risk of fever and changed the serum cytokine profile to be more similar to breastfed infants in a nonrandomized comparison group; osteopontin supplementation did not affect infant growth (64).

There have been dozens of trials assessing nutrient supplementation of MOM or donor human milk to improve growth in low-birth-weight and preterm infants; the majority have been conducted in very-low-birth-weight infants (<1500 g) and all have been conducted in high-income countries. Protein and energy supplementation of both human milk and formula increases weight gain, linear growth, and head growth among preterm infants (65). Reviews assessing high versus low protein supplementation in preterm infants also suggest growth benefits of higher protein (66). Trials assessing supplementation of LC-PUFAs to infant milk show no benefits on growth in preterm infants (67). There are several amino acid-fortification trials. Taurine fortification of formula has been linked to reduced linear growth (68). As reported in a meta-analysis, one glutamine fortification trial found that it improved infant growth, but the results were not replicated in 2 other studies (69).

## Advances and Technologies for Human-Milk Analysis

Human milk is chemically and physically a complex biofluid containing relatively high amounts of lipids and carbohydrates, and numerous living cells and organisms. Due to these complexities, human-milk analysis has been challenging, requiring adjustments in sample preprocessing and assay validation and optimization. More effort has recently been invested in evaluating existing serum- or plasma-based methods for nutrient analysis for their suitability for milk (70–73). New opportunities also exist to comprehensively characterize human-milk composition with rapidly evolving ’omics technologies. Applications of the modern technologies in human-milk research, coupled with the optimal use of conventional analytical methods, provide a powerful means to understand human milk as a biological system.

### Macro- and micronutrients

Conventional methods for macro- and micronutrient analysis of human milk have been validated and described previously (71, 74). Recent developments in technologies and instruments have enabled sensitive, rapid, and simultaneous analyses of selective nutrients. For example, ultra-high-performance LC–tandem MS (UHPLC–MS/MS) and inductively coupled plasma–atomic emission spectrometry coupled with a mass spectrometer (ICP–MS) are now optimized for the analysis of a panel of B vitamins (vitamin B-1, B-2, B-3, and B-6) and minerals (iron, copper, zinc, iodine, calcium, and magnesium), respectively (75, 76). Also, a human-milk analyzer based on mid- and near-infrared spectroscopy has been developed to simultaneously measure all macronutrients (77, 78).

### Bioactives

Human-milk metabolomics is an emerging discipline that offers an opportunity for insights into the chemical interactions between the maternal-infant-milk compartments. The commonly used analytical platforms are NMR, GC, or LC coupled with MS (79). Among MS-based metabolomics, a commercially available targeted metabolomics assay (AbsoluteIDQ® p180, Biocrates kit) has been validated for milk by LC–tandem MS (LC–MS/MS) (80). The global profiling of the human-milk metabolome with untargeted metabolomics platforms has aided identifying metabolites originating from mothers, micro-organisms, and other exogenous chemicals (81). Recent application of MS-based proteomics to human-milk research has led to systematic characterization of a milk proteome. The human-milk proteome encompasses thousands of proteins, including enzymes, glycoproteins, and endogenous peptides that play an important role in infant growth and the development of the gastrointestinal tract, the immune system, and the brain (82). Proteomic analysis of human-milk-derived exosomes has revealed a unique set of functional proteins (83, 84). To facilitate proteomic discovery of low-abundance proteins, additional preprocessing and protein fractionation are recommended to enhance their resolution (85). DNA aptamer-based proteomics appears to be a potential platform, although it requires validation and optimization for milk (86). For milk immunoglobulins, a proteome microarray has been used to characterize complex immune responses (IgA and IgG) to multiple pathogens, demonstrating a potential mechanism underlying the protective effects of human milk (87).

### Milk microbiome

Our knowledge of the human-milk microbiome has increased exponentially in the past decade (88); however, the fate and function of microbes in milk are not well understood. Culture-dependent methods and culture-independent technologies (quantitative PCR, denaturing gradient gel electrophoresis, and 16S rRNA) provide complementary views of the unique, rich bacterial community in human milk (89). High-throughput platforms such as next-generation sequencing, which uses total microbial DNA (metagenomics), can enhance the resolution of taxonomic assignments and provide information on their metabolic potential (90). In addition to metagenomics, integrative meta-omics approaches, such as meta-transcriptomics, meta-proteomics, and metabolomics, have evolved and been applied to translational microbiome research (91). The application of multi-omics strategies in human-milk research will improve our understanding of not only descriptive bacterial communities’ composition but also their functions in concert with other milk components to impact infant health in the human-milk ecosystem.

### Considerations in new methods of human-milk sampling and analyses

Human-milk composition varies depending on stage of lactation, time of the day, and feeding stage. Once samples are collected, sample handling (e.g., freeze–thaw cycle), storage (e.g., temperature), and preprocessing (e.g., pasteurization) can affect the composition. Thus, standardized, validated sampling and handling protocols are important to control sample variability and quality (70). Dried milk spot and microsampling systems, including volumetric absorptive microsampling, are being adapted to study human-milk metabolomics (92, 93), facilitating practical and field-friendly collection, especially in remote settings. However, analyte throughput and stability and accuracy and reliability of quantification are major challenges.

Major challenges and considerations also exist in applying ’omics technologies to human-milk research. Untargeted ’omics analysis is based on the qualitative identification and relative quantification of biological molecules to generate hypotheses related to newly identified components and their functions in the human-milk system. Targeted validation and absolute quantification, however, are warranted to test ’omics-derived hypotheses to generate biologically robust evidence. Well-designed studies and experiments (e.g., longitudinal sampling and a randomized controlled dose–response trial), along with a well-characterized population (e.g., metadata on maternal and infant nutrition and health and other key factors that influence milk composition) (94) and focused hypotheses to address key biological questions, are important next steps. In addition, the use of standard operating procedures as well as cross-laboratory and cross-platform validations would enhance reproducibility. Rigorous bioinformatics and mathematical tools for data integration, interpretation, visualization, and reconstruction are also needed to fully mine and gain biologically meaningful insights from high-dimensional human-milk ’omics data.

## Ongoing Research

We have laid out a detailed research agenda of 2 funding agencies to not only stimulate new research but also to extend the value of ongoing efforts. The Maternal, Newborn Child Health Discovery & Tools team at the Bill & Melinda Gates Foundation aims to understand human-milk composition as an important part of its strategy to promote lactating women's health, and support optimal growth and thriving of children in the community. The foundation funds research projects that support and facilitate the discovery, synthesis, and application of evidence-based knowledge of human milk. Prioritized research questions include the following: *1*) understanding variability in human-milk composition in different geographical settings and its role as an important mediator of associations between maternal and infant nutrition and health, *2*) establishing global reference values for nutrients and bioactives in human milk from healthy lactating women, and *3*) evaluating effects of maternal nutrient supplementation during lactation on human-milk composition. The ultimate goals of these investments are to fill the knowledge gaps in human-milk composition and inform maternal and infant nutrition interventions and global health policies regarding maternal nutrition during lactation.

The US NIH, led by the *Eunice Kennedy Shriver* National Institute of Child Health and Human Development (NICHD), has a long history of funding research pertaining to breastfeeding, lactation, and human milk (https://report.nih.gov/categorical_spending.aspx). This NIH-supported research includes work across the entire spectrum of research from basic physiology and mechanisms, through animal models, to clinical trials and behavioral interventions, then to dissemination and implementation science. This research covers a range of topics including milk composition, breastfeeding behaviors, pharmacology, lactation physiology, infectious disease transfer, microbiome, hormones, obesity, breast cancer risk, and many other topics. Of particular interest, notably and apropos of the objectives of this perspective, to date the vast majority of the research focused on human-milk composition has been focused primarily on the effect of single-nutritive or nonnutritive components in milk, with very few investigators approaching the interrogation of human milk as a biological system.

With the current ability to use multi-omics tools and improved statistical modeling techniques, there is an opportunity to better understand this critical aspect of human biology. The importance of human-milk research has also been emphasized in both the NICHD strategic plan (https://www.nichd.nih.gov/about/org/strategicplan) and the recent trans-NIH Strategic Plan for NIH Nutrition Research (https://www.niddk.nih.gov/about-niddk/strategic-plans-reports/strategic-plan-nih-nutrition-research).

Importantly, many other organizations (such as the March of Dimes, the Gerber Foundation, and the Family Larsson-Rosenquist Foundation) also invest in human-milk research. Many for-profit entities, including but not limited to infant formula companies, invest in this space as well, demonstrating the importance of this research to the public and private sectors alike and the potential of broad partnerships.

To emphasize the importance of the issues highlighted in this perspective, and to encourage nutritional, biological, and social science research focusing on understanding human milk as a biological system, NICHD has started the “Breastmilk Ecology: Genesis of Infant Nutrition” (BEGIN) Initiative. The objective of BEGIN is to expand our understanding of the components and functional implications of human milk as a biological system and its ecology. BEGIN will specifically address the following themes: *1*) mechanism and role of maternal factors influencing lactation and human-milk composition; *2*) understanding of human-milk composition and its components and their interactions within the matrix; *3*) infant factors influencing human-milk composition, volume, and lactation performance over time; *4*) how to utilize emerging technologies (e.g., artificial intelligence, ’omics, etc.) to better understand human-milk biology and its functional implications; and *5*) understanding how the integration of this emerging knowledge will impact on messaging regarding infant feeding choice and practices. Ultimately, these efforts will identify and make available the information and evidence needed for the development of nutritional guidance, reference standards, and recommendations to address many of the research gaps highlighted in this perspective.

## Conclusions

We have much to learn about human milk. Could human milk be an exemplar of precision nutrition and specifically produced by the mother for her infant? Could a mother's own milk be tailored to the needs of her growing baby? Could the suckling of the infant at the mother's breast be a conduit for the communication of analytes between the infant's oral cavity and the mother's mammary gland—a form of intended bidirectional signaling? Or, is it possible that a mother's milk composition is the result of the combination of genetics and environment that is not necessarily matched to the individual needs of her baby? Unfortunately, we still do not know the answer to those questions. However, understanding this mother-milk-infant “triad” (95) provides opportunities to vastly improve maternal–child health; as such, scientists should interrogate human milk moving forward using precision nutrition tools including systems biology, artificial intelligence, and other advanced analytical approaches.

## Notes

The authors report no funding received for this work. The contents of this article represent the authors’ views and do not constitute an official position of the NIH or the US government.

Abbreviations used: BEGIN, Breastmilk Ecology: Genesis of Infant Nutrition; HMO, human-milk oligosaccharide; LC-PUFA, long-chain PUFA; MOM, mother's own milk; NICHD, National Institute of Child Health and Human Development.
